# Protective Role of Glutathione in the Hippocampus after Brain Ischemia

**DOI:** 10.3390/ijms22157765

**Published:** 2021-07-21

**Authors:** Youichirou Higashi, Takaaki Aratake, Takahiro Shimizu, Shogo Shimizu, Motoaki Saito

**Affiliations:** Department of Pharmacology, Kochi Medical School, Kochi University, Kohasu, Okoh-cho, Nankoku 783-8505, Japan; higasi@kochi-u.ac.jp (Y.H.); b18d6b01@s.kochi-u.ac.jp (T.A.); shimizu@kochi-u.ac.jp (T.S.); shimizu-sh@kochi-u.ac.jp (S.S.)

**Keywords:** glutathione, ischemia, hippocampus, zinc, oxidative stress, excitatory amino acid carrier 1

## Abstract

Stroke is a major cause of death worldwide, leading to serious disability. Post-ischemic injury, especially in the cerebral ischemia-prone hippocampus, is a serious problem, as it contributes to vascular dementia. Many studies have shown that in the hippocampus, ischemia/reperfusion induces neuronal death through oxidative stress and neuronal zinc (Zn^2+^) dyshomeostasis. Glutathione (GSH) plays an important role in protecting neurons against oxidative stress as a major intracellular antioxidant. In addition, the thiol group of GSH can function as a principal Zn^2+^ chelator for the maintenance of Zn^2+^ homeostasis in neurons. These lines of evidence suggest that neuronal GSH levels could be a key factor in post-stroke neuronal survival. In neurons, excitatory amino acid carrier 1 (EAAC1) is involved in the influx of cysteine, and intracellular cysteine is the rate-limiting substrate for the synthesis of GSH. Recently, several studies have indicated that cysteine uptake through EAAC1 suppresses ischemia-induced neuronal death via the promotion of hippocampal GSH synthesis in ischemic animal models. In this article, we aimed to review and describe the role of GSH in hippocampal neuroprotection after ischemia/reperfusion, focusing on EAAC1.

## 1. Introduction

Stroke is one of the most frequent causes of death worldwide and a major cause of serious disability. Lifestyle factors that have been shown to increase the risk of stroke include smoking; lack of physical activity; being overweight; and increased intake of salt, alcohol, and fat. These factors also impact on outcomes in patients with stroke. According to a systematic analysis from 2016, there were 80.1 million prevalent cases of stroke, 5.5 million deaths, and 116.4 million disability-adjusted life years resulting from stroke globally that year [1]. In recent years, although the rate of deaths due to acute ischemic stroke has declined because of the introduction of new therapeutic strategies and specialized care, ischemic stroke survivors continue to suffer from disabilities [2]. In particular, ischemia-induced damage to the hippocampus, the main brain structure related to learning and memory [3], is recognized as a serious problem, as it leads to cognitive impairment [4].

The pathophysiological mechanisms underlying ischemia-induced hippocampal damage have been studied intensively. Oxidative stress, a hallmark of brain ischemia, has been identified as a major factor. It results from the increased generation of reactive oxygen species (ROS) and reactive nitrogen species, which lead to apoptosis and necrosis of neurons [5,6]. Another factor is the disruption of zinc (Zn^2+^) homeostasis in the hippocampus. Zn^2+^ levels are maintained at a constant level in the adult mammalian brain, including the hippocampus, in which Zn^2+^ participates in normal physiological brain functions, such as learning and memory [7]. However, ischemia/reperfusion disrupts neuronal Zn^2+^ homeostasis, resulting in neuronal cell death within the hippocampus [8]. Therefore, not only oxidative stress but also the disruption of neuronal Zn^2+^ homeostasis are recognized as important therapeutic targets for the treatment of ischemia-induced hippocampal injury.

Glutathione (GSH) is the most abundant low molecular weight thiol compound within cells, and it plays a crucial role in cell defense not only against oxidative stress but also against the disruption of Zn^2+^ homeostasis. In neurons, GSH levels are predominantly regulated by excitatory amino acid carrier 1 (EAAC1), which can transport cysteine, a substrate for GSH synthesis. Accumulating evidence indicates that EAAC1 gene deletion exacerbates ischemia-induced hippocampal damage via impaired Zn^2+^ homeostasis as well as oxidative stress [9], and that diurnal fluctuations in EAAC1 levels affect susceptibility to ischemia-induced neuronal cell death in the hippocampus [10]. These findings suggest that neuronal GSH levels regulated by EAAC1 are crucial in this context. In this review, we focus on EAAC1 expression in the hippocampus and discuss the role of GSH in regulating oxidative stress and zinc homeostasis, which are primary pathological findings following brain ischemia. Furthermore, we describe how EAAC1 was demonstrated to orchestrate GSH levels, thus conferring neuroprotection within the hippocampus, and how these molecules are subject to the circadian rhythm.

## 2. Protective Roles of Glutathione (GSH) in Ischemia-Induced Hippocampal Injury

### 2.1. Anti-Oxidative Role of GSH in Ischemia-Induced Oxidative Stress

Cerebral ischemia leads to a marked increase in oxidative stress resulting from the generation of ROS, such as superoxide, hydrogen peroxide, and hydroxyl radicals [5]. Oxidative stress can rapidly damage many components of the cell, including lipids, proteins, and DNA, whereby it contributes to the progression of neuronal cell death after ischemic stroke [11,12,13]. Therefore, oxidative stress is recognized as one of the major mediators of cerebral ischemia/reperfusion-induced hippocampal injury [6,14,15].

**Figure 1:**
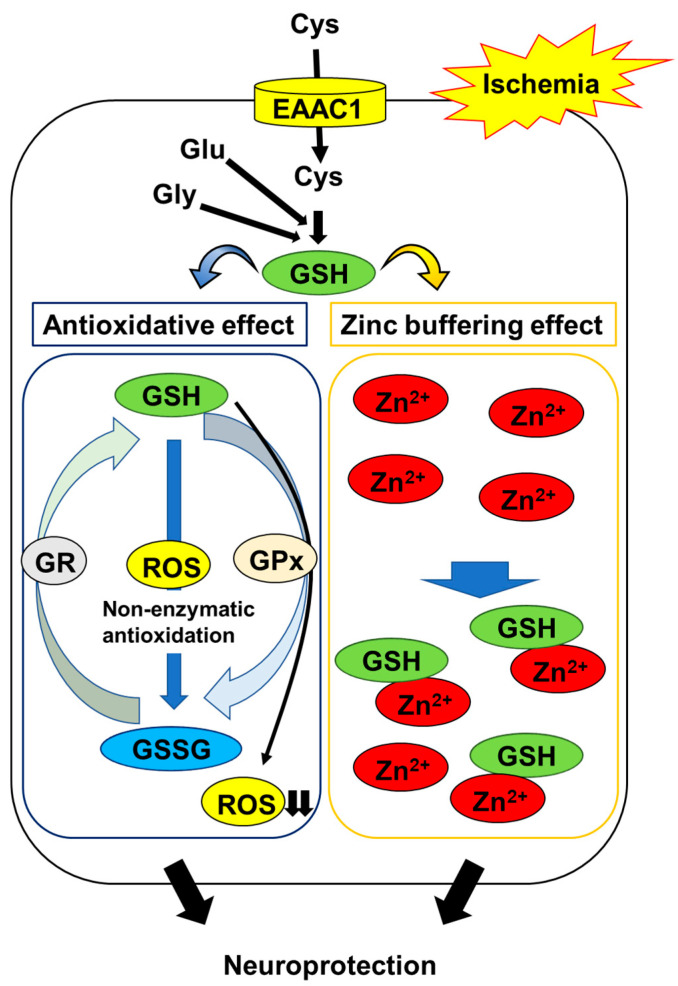
Mechanisms of GSH protection against hippocampal neuron damage following brain ischemia. The abbreviations are as follows: cysteine (Cys), glutamate (Glu), glycine (Gly), excitatory amino acid carrier 1 (EAAC1), glutathione (GSH), glutathione disulfide (GSSG), GSH reductase (GR), GSH peroxidase (GPx), reactive oxygen species (ROS).

GSH is a major endogenous component of the cellular antioxidant defense. It is capable of scavenging various ROS directly. Research has demonstrated that the order of GSH reactivity towards the radicals is ^•^OH > ^•^OCH_3_ > ^•^OOH > ^•^OOCCl_3_ > ^•^OOCHCH_2_ > ^•^OOCH_3_, and that the rate constants range from 2.02 × 10^4^ M^−1^ s^−1^ to the diffusion limit (7.68 × 10^9^ M^−1^ s^−1^) [16]. These findings indicate that GSH is an excellent free radical scavenger. On the other hand, GSH is also known to act as a substrate for a number of glutathione peroxidases to detoxify ROS [17]. During these reactions, GSH is oxidized and converted into glutathione disulfide (GSSG), which is subsequently reduced to GSH by glutathione reductase [18]. However, cellular GSH levels can be lowered by the export of GSSG when the production of the latter exceeds the capacity for recycling to the former, indicating that this antioxidative defense system cannot offset oxidative stress [19,20]. Indeed, many studies have demonstrated that the brain content of endogenous GSH is depleted during ischemia/reperfusion [21,22,23], and that treatment with GSH or glutathione monoethyl ester, a cell-permeable derivative of GSH, ameliorates ischemia-induced oxidative damage, including DNA and lipid oxidation in the hippocampus [24,25]. These observations suggest that maintaining neuronal GSH levels is an important constituent in protecting neurons against oxidative stress in the post-ischemic hippocampus (Figure 1).

Interestingly, Won et al. demonstrated that the GSH levels in CA1 pyramidal neurons were decreased during the first few hours of ischemia/reperfusion, accompanied by increased superoxide levels; conversely, the prevention of ischemia/reperfusion-induced increase in superoxide production using an inhibitor of nicotinamide adenine dinucleotide 3-phosphate oxidase, a major source of ROS, was found to suppress the decline in GSH in post-ischemic neurons [26]. On the other hand, they also showed that mice in which neuronal GSH levels were maintained by treatment with N-acetyl cysteine (NAC), a cell-permeable precursor of GSH, exhibited a reduction in neuronal oxidative stress and neuronal cell death in the hippocampus following ischemia/reperfusion [26]. These findings indicate that the depletion of neuronal GSH is both a result and a cause of neuronal oxidative stress following ischemia/reperfusion. In contrast, changes in the levels of GSH in post-ischemic glial cells, such as astrocytes and microglia, remain to be elucidated. Astrocytes play an important role in the synthesis of neuronal GSH by supplying precursor molecules [27]. Therefore, it is necessary to clarify the role of glial GSH in neuronal survival in the post-ischemic hippocampus.

### 2.2. GSH Protects Neurons from Ischemia-Induced Disruption of Intracellular Zn^2+^ Homeostasis

Zn^2+^ is involved in hippocampal injury following brain ischemia. In the mammalian brain, most of the Zn^2+^ is bound to specialized Zn^2+^ proteins—for example, metallothionein and transcription factors [28]. A smaller part of it is concentrated in the presynaptic vesicles in a subset of glutamatergic neurons throughout the forebrain, especially in the hippocampus [29,30,31]. Therefore, the intracellular free Zn^2+^ concentration in the hippocampus is considered to be very low under normal physiological conditions [28]. However, the stored Zn^2+^, along with the protein-bound portion, is massively released during ischemia and the onset of reperfusion, subsequently accumulating within hippocampal neurons [32,33,34,35,36,37,38]. The prevention of this process using Zn^2+^ chelators, including calcium disodium ethylenediamine tetraacetate and clioquinol, has been shown to protect hippocampal neurons from ischemia-induced neurodegeneration [8,39], suggesting that the ischemia-induced disruption of neuronal Zn^2+^ homeostasis contributes to neurodegeneration in the hippocampus.

The thiol moiety of GSH has a high affinity for Zn^2+^ through non-enzymatic conjugation. Treatment with GSH protects neurons against Zn^2+^ accumulation-induced cell toxicity [40]. The replenishment of GSH using NAC reduces Zn^2+^ accumulation and neurodegeneration in the post-ischemic hippocampus [41]. These findings suggest that GSH acts as an intrinsic factor that buffers intracellular Zn^2+^ concentrations to prevent the ischemia-induced disruption of Zn^2+^ homeostasis; in addition to its antioxidative activity, this mechanism protects neurons (Figure 1).

In contrast, Zn^2+^ binds and inhibits glutathione reductase and peroxidase, the major enzymes of the glutathione redox cycle [42,43]. Chen and Liao demonstrated evidence indicating that Zn^2+^ entry into neurons causes a decrease in intracellular GSH levels and eventually triggers neuronal cell death [40]. GSH can suppress the ischemia-induced disruption of Zn^2+^ homeostasis, but this function may be affected by the accumulation of Zn^2+^ in neurons.

## 3. Neuroprotective Effects of EAAC1

EAAC1, a member of the sodium-dependent excitatory amino acid transporter (EAAT) family, is localized on neuronal plasma membranes and is highly abundant in the cerebral cortex, hippocampus, and striatum [44,45,46]. EAAC1 was originally identified as a neuronal high-affinity glutamate transporter [47]. Although research has shown that it contributes only modestly to the clearance of glutamate from the synaptic space, which is primarily performed by astrocytic glutamate transporters, such as glutamate-aspartate transporter and glutamate transporter 1 [48,49], accumulating evidence indicates that EAAC1 can bind to extracellular cysteine and mediate its transport into presynaptic terminals or postsynaptic dendrites more effectively than other EAATs can [50,51,52,53,54]. Neurons in the hippocampus of EAAC1-deficient mice exhibit decreased GSH levels, increased oxidant levels, and vulnerability to oxidative stress. These changes are abrogated by treating the animals with NAC, suggesting that EAAC1 is the major route for neuronal cysteine uptake and contributes to neuronal GSH synthesis [9,55].

Recently, the role of EAAC1 in neuronal resistance to ischemia was demonstrated. EAAC1-deficient mice subjected to transient cerebral ischemia were found to exhibit exacerbated neuronal injury in the hippocampus and cortex and a corresponding increase in ROS production [9,56,57]. Furthermore, it was revealed that EAAC1 gene deletion results in an increase in the basal levels of cytosolic and vesicular Zn^2+^ in hippocampal and cortical neurons and causes an elevation in ischemia-induced Zn^2+^ accumulation. The treatment of EAAC1-deficient mice with Zn^2+^chelators or NAC reduces ischemia-induced neuronal injury, ROS production, and Zn^2+^ accumulation [9,56]. EAAC1 expression was demonstrated to be increased in the hippocampus and cortex as early as 8 h after transient forebrain ischemia [58]. Together, these findings suggest that EAAC1-dependent GSH synthesis via cysteine uptake is one of the major defense mechanisms against neuronal injury in the hippocampus following brain ischemia.

In addition, the loss of the EAAC1 gene has been shown to evoke other pathological alterations in the post-ischemic hippocampus. One example is adult hippocampal neurogenesis. Over the last few decades, it has become clear that this process, which originates from neural progenitor cells, occurs continuously in the subgranular zone of the dentate gyrus [59], and newly formed neurons functionally integrate into the existing neural circuitry within the hippocampus [60]. Several studies have shown that the proliferation of neural progenitor cells is increased in the subgranular zone and peaks around 7 days following ischemic injury, suggesting its potential role in neural repair after stroke [61,62,63]. However, Choi et al. found that EAAC1 was expressed in mature and immature neurons in the subgranular zone and that EAAC1-deficient mice exhibited a decrease in their overall level of neurogenesis, including cell proliferation, neuronal differentiation, and survival after ischemia, as compared with wild-type controls [64]. Another pathological alteration in post-ischemic EAAC1-deficient mice is the integrity of the blood–brain barrier. The ischemia-induced leakage of serum IgG into the hippocampal parenchyma and disorganization of the diameter and density of the blood vessels were found to be aggravated in EAAC1-deficient mice; these changes were suppressed by NAC treatment [57]. Given that EAAC1 is primarily expressed in mature and immature neurons, these findings suggest that EAAC1-dependent GSH synthesis functions as a direct and/or indirect protective system against ischemic brain injury. However, further studies are needed to determine how EAAC1-mediated GSH synthesis in neurons can protect blood–brain barrier integrity.

## 4. Neuroprotective Role of GSH and Time-of-Day Variations in Ischemic Injury

Circadian rhythms play an important role in the regulation of many biochemical, physiological, and behavioral functions. Accumulating evidence indicates that stroke occurrence follows circadian rhythms, with a higher frequency in the morning (06:00–12:00) [65,66,67,68,69], which is mediated by diurnal variations in blood pressure, body temperature, and blood coagulation [65,70,71]. In contrast, several studies examining the daily variation in the susceptibility of the brain to ischemic stroke yielded different results. They revealed that the mortality rate associated with ischemic stroke is higher during sleep at night than during wakefulness [71,72], although the underlying mechanisms remain unclear.

Recently, using a mouse model of ischemic stroke, Beker et al. demonstrated that transient brain ischemia at night resulted in reduced neuronal cell death, brain swelling, and neurological deficit scores, and that the expression of circadian proteins was altered in response to ischemic injury [73]. Furthermore, mice subjected to transient global ischemia during the light phase have greater numbers of degenerating hippocampal neurons [74]. Considering that mice are nocturnal, these findings suggest that the daily variation in the susceptibility of the brain to ischemic stroke in mice is correlated with that in humans.

**Figure 2:**
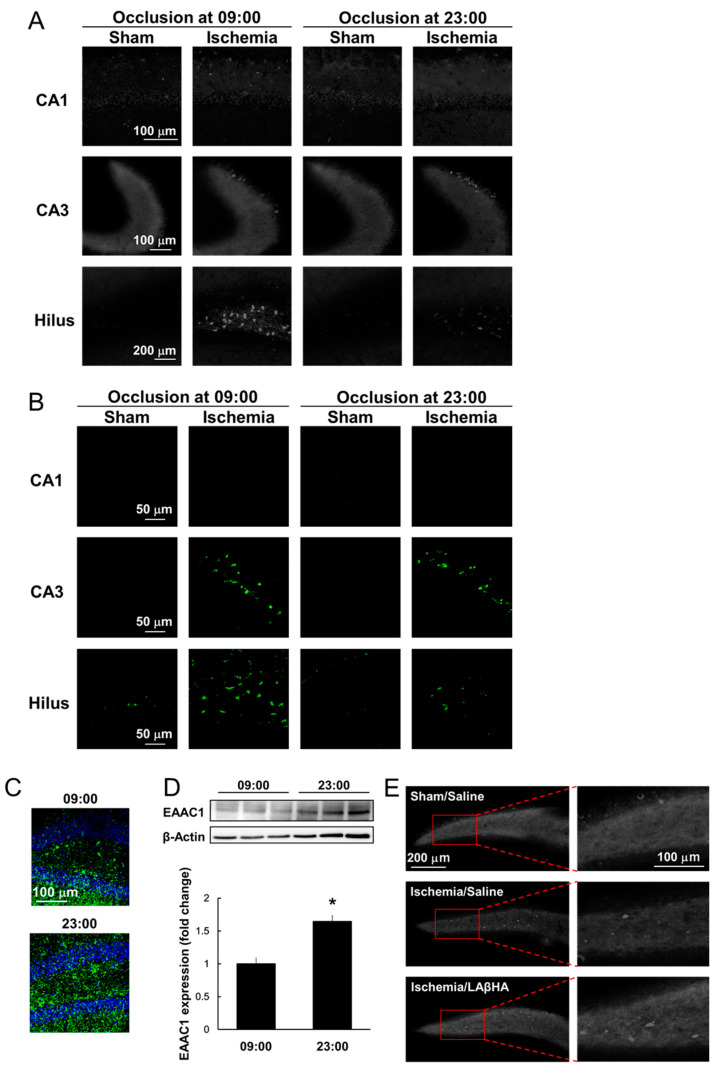
Reduced ischemia-induced Zn^2+^ accumulation and neuronal injury in response to increased GSH levels via elevated EAAC1 protein expression at 23:00. (**A**) Representative images showing TSQ fluorescence (indicative of Zn^2+^ accumulation) in the hippocampus 72 h after transient brain ischemia. Zn^2+^ accumulation was observed in the CA3 and hilar but not the CA1 region in ischemic mice. Notably, the hilar Zn^2+^ accumulation was remarkably reduced in mice subjected to transient brain ischemia at 23:00 compared to that at 09:00. (**B**) Fluorescent images showing neurodegeneration by Fluoro-Jade B staining in the hippocampus 72 h after transient brain ischemia in the CA3 and hilar but not in the CA1 region in ischemic mice. In the hilus, Fluoro-Jade B fluorescent signals were remarkably reduced in mice subjected to transient brain ischemia at 23:00 compared to those at 09:00. (**C**) Representative images depicting double immunofluorescence staining for glutathione adduct with N-ethylmaleimide (GS-NEM, represents GSH levels; green) and DAPI (blue) in the hilar region. GSH levels were significantly higher in the hilus at 23:00 than at 09:00. (**D**) EAAC1 protein expression was analyzed by Western blotting. EAAC1 protein levels in the hippocampus were significantly increased at 23:00 compared to at 09:00. * *p* < 0.05, relative to the hippocampus harvested at 09:00 (Mann-Whitney U test). (**E**) Representative images highlighting TSQ fluorescence in the hilar region 72 h after ischemia in mice pretreated with L-aspartic acid β-hydroxamate (LAβHA), a selective EAAC1 inhibitor, at 23:00. Ischemia-induced Zn^2+^ accumulation was significantly increased in LAβHA-pretreated mice.

Our recent study demonstrated that ischemia-induced Zn^2+^ accumulation and neuronal cell death vary with the time of day in the hippocampus in mice. When transient global ischemia was induced by clipping the bilateral common carotid arteries at two different time points (at 09:00, 4 h after the beginning of the light phase, and at 23:00, 4 h after the beginning of the dark phase), dense intracellular Zn^2+^ accumulation was observed in the hippocampal CA3 and hilar regions, but not in CA1 (Figure 2A). Mice subjected to global ischemia at 23:00 had remarkably less Zn^2+^ accumulation in the hilus than those manipulated at 09:00 (Figure 2A). Interestingly, mice subjected to transient global ischemia at 23:00 also exhibited a significant decrease in the number of degenerating neurons in the hilus compared to their 09:00 counterparts (Figure 2B). Accumulating evidence suggests that intracellular Zn^2+^ accumulation is a trigger for neuronal death with features of necrosis and apoptosis in cell cultures and in the brain after transient ischemia [75,76]. These findings provide evidence that Zn^2+^ accumulation induced by transient global ischemia is a crucial part of the time-of-day variation in ischemia-induced hilar neurodegeneration.

Interestingly, the EAAC1 protein levels exhibit a diurnal variation, peaking in the mouse mesencephalon during the night; this is regulated by post-translational mechanisms via microRNA miR-96-5p [77]. Given that GSH is involved in the maintenance of Zn^2+^ homeostasis in neurons, it can be hypothesized that neural levels of GSH are subject to the time-of-day variation in ischemia-induced hilar neurodegeneration. Indeed, the hilar levels of GSH were found to be higher in normal mice at 23:00 than at 09:00 (Figure 2C) and, intriguingly, the EAAC1 protein expression followed a similar pattern (Figure 2D). The treatment of mice with a selective EAAC1 inhibitor before ischemia at 23:00 resulted in aggravated Zn^2+^ accumulation in the hilus (Figure 2E). These findings suggest that increased EAAC1 expression and GSH synthesis in the hilus of mice at 23:00 may confer tolerance to transient global ischemia via an increase in the hilar GSH content [10] (Figure 2). On the other hand, abnormal protein expression and activity of glutathione peroxidase were observed in the brains of mice with age-related neurodegenerative diseases such as Alzheimer’s disease, frontotemporal dementia, and Parkinson’s disease [78]. Neuronal cysteine uptake by EAAC1 was inhibited by soluble amyloid oligomers [79], and aberrant EAAC1 accumulations were observed in the hippocampal neurons of Alzheimer’s disease patients [80]. It seems that neuronal aging may participate in the dysfunction of GSH metabolism in the hippocampus via altered glutathione peroxidases and EAAC1. Recently, it was revealed that aging modifies the temporal pattern of glutathione peroxidase expression and activity in the hippocampus [81]. Taken together with that the prevalence of stroke in elderly patients has been increasing in recent years; therefore, investigating whether neuronal aging affects the time-of-day variation in ischemia-induced hilar neurodegeneration is expected.

## 5. Conclusions

Neuronal GSH plays an important role in protecting neurons from cell damage after transient global brain ischemia by ameliorating hippocampal oxidative stress and the disruption of Zn^2+^ homeostasis. However, transient global ischemia results in decreased levels of neuronal GSH in the hippocampus. While this decrease is caused by oxidative stress and accumulated Zn^2+^, it is thought that the ischemia-induced decrease in neuronal GSH causes further increases in ROS production and the disruption of Zn^2+^ homeostasis, eventually leading to neuronal cell death and the impairment of hippocampal functions. In addition, EAAC1 is responsible for the neuronal supply of cysteine, which is the rate-limiting substrate for GSH synthesis. The expression levels of EAAC1, regulated by diurnal fluctuations in the hippocampus, affect susceptibility to ischemic neuronal injury. Therefore, the maintenance of intracellular GSH levels via EAAC1 is essential for neuronal survival after brain ischemia and may be an important therapeutic target.
